# The use of budesonide in IgA pediatric patients with recurrent macroscopic hematuria: a single-center real-life experience

**DOI:** 10.1093/ckj/sfaf109

**Published:** 2025-04-12

**Authors:** Luigi Annicchiarico Petruzzelli, Oriana De Marco, Gabriele Malgieri, Eleonora Riccio, Antonio Pisani

**Affiliations:** Department of Pediatric Specialties, Pediatric Nephrology, Dialysis and Renal Transplantation Santobono Pausilipon Children's Hospital, Naples, Italy; Chair of Nephrology, Department of Public Health, University Federico II of Naples, Naples, Italy; Department of Pediatric Specialties, Pediatric Nephrology, Dialysis and Renal Transplantation Santobono Pausilipon Children's Hospital, Naples, Italy; Chair of Nephrology, Department of Public Health, University Federico II of Naples, Naples, Italy; Chair of Nephrology, Department of Public Health, University Federico II of Naples, Naples, Italy

To the Editor,

Immunoglobulin A nephropathy (IgAN) is the most common primary glomerulonephritis worldwide, with a heterogeneous clinical presentation ranging from isolated hematuria to nephrotic syndrome and progressive renal insufficiency [1]. Current treatment strategies emphasize supportive care with renin–angiotensin system inhibitors, but patients with frequent macroscopic hematuria (MH) episodes and significant proteinuria often require additional immunosuppressive therapy [2]. Budesonide, a second-generation corticosteroid, exerts a targeted effect on Peyer's patches in the ileum, where IgA production is initiated. Despite its demonstrated efficacy in adult populations, data on pediatric patients remain scarce [3].

Here we report a retrospective, single-center study between 2023 and 2024, evaluating the efficacy and safety of budesonide in pediatric patients with IgAN who presented with recurrent MH. A total of eight pediatric patients (five males, three females; median age: 11.1 years) with recurrent macroscopic hematuria were included in this study. All patients had a history of at least one episode of macroscopic hematuria per year before treatment, with some experiencing up to seven episodes annually. All patients had previously received corticosteroids or immunosuppressive agents without sustained remission. Budesonide was administered as a second-line therapy at a dose of 9 mg/m^2^/day for 4 weeks, followed by alternate-day dosing, tailored to individual clinical responses, for a total duration of 12 months.

Our patients achieved complete resolution of MH episodes following budesonide therapy. Before treatment, annual MH recurrence ranged from one to seven episodes, with the longest episode-free period being 2 months. At Month 12, all patients remained MH-free, and reductions in proteinuria levels were observed (Fig. 1). Renal function remained unchanged, and there were no complications or side effects. (Supplementary data, Table S1).

**Figure 1:**
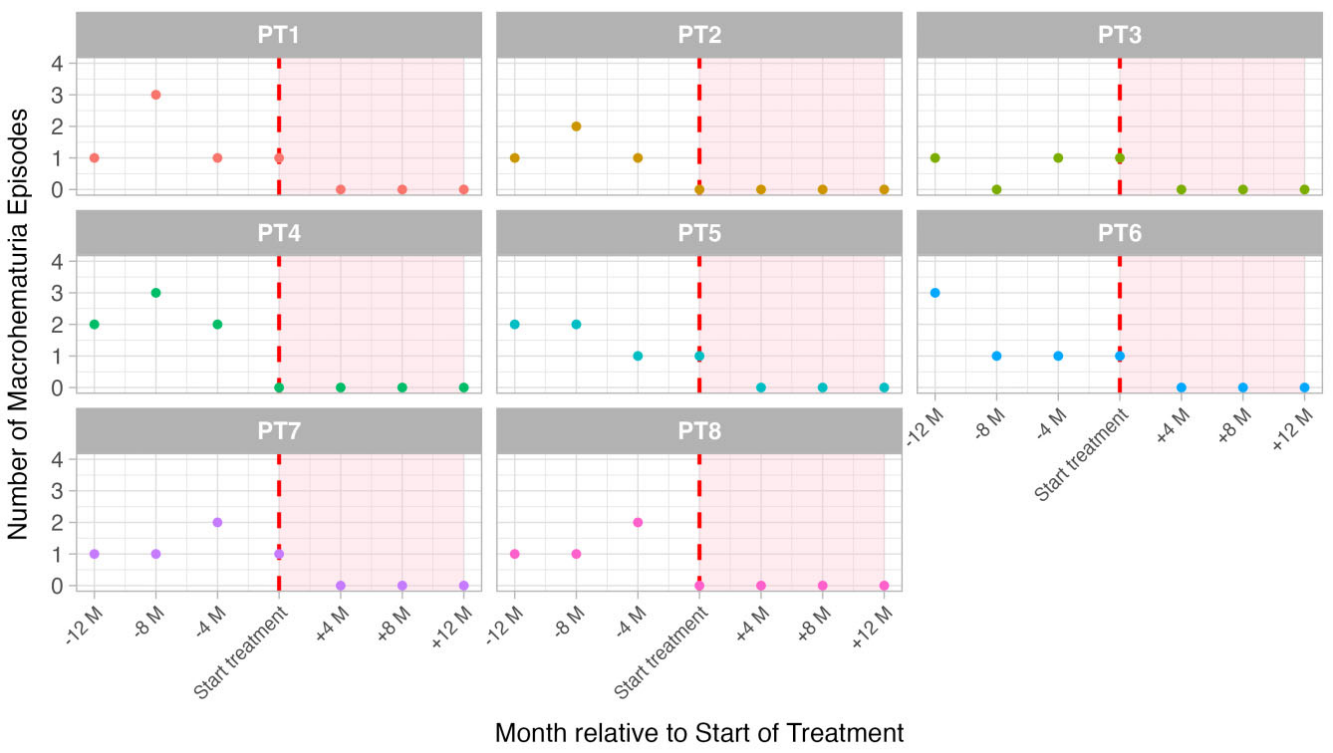
Distribution of MH episodes before and after budesonide treatment. M, months. Pink-shaded area indicates treatment period.

The role of hematuria in IgAN is often overlooked, despite evidence linking persistent microscopic hematuria to glomerular inflammation, complement activation and renal injury progression [4].

Our findings indicate that budesonide may serve as an effective alternative therapy for pediatric patients with recurrent MH and proteinuria, providing prolonged reduction of MH and reducing reliance on systemic corticosteroids.

The NEFIGAN (NCT01738035) and NEFIGARD (NCT03643965) trials previously demonstrated the efficacy of targeted-release budesonide (Nefecon) in reducing proteinuria and stabilizing renal function in adults with IgAN [3, 5]. However, pediatric-specific data remain limited. Our study supports the potential utility of budesonide in this population, though larger prospective studies are warranted to validate these findings and establish standardized dosing protocols.

## Supplementary Material

sfaf109_Supplemental_File
