# Supplementary material for: The use of budesonide in IgA pediatric patients with recurrent macroscopic hematuria: a single-center real-life experience
Source: Clin Kidney J. 2025 Apr 12;18(5):sfaf109. doi: 10.1093/ckj/sfaf109 (PMC12046507; doi:10.1093/ckj/sfaf109)
Supplement: sfaf109_Supplemental_File [file sfaf109_supplemental_file.docx]

Supplementary Table 1. Clinical characteristics of patients at baseline and after Budesonide therapy

| Patient | Age at diagnosis (years) | Gender | Renal biopsy pathology | Previous immunosuppressant | Episodes of MH per year before Budesonide | eGFR(ml/min/1.73m2) before Budesonide | Serum creatinine(mg/dl) before Budesonide | 24h urinary protein(g/24h) before Budesonide | Response before Budesonide | Status at last follow-up | Complications of Budesonide therapy | eGFR(ml/min/1.73m2) after Budesonide at last follow-up | Serum creatinine(mg/dl) after Budesonide at last follow-up | 24h urinary protein(g/24h) after Budesonide at last follow-up |
| --- | --- | --- | --- | --- | --- | --- | --- | --- | --- | --- | --- | --- | --- | --- |
| PT1 | 15 | F | M1E1S0T0C1 | CS, MMF | 6 | 90 | 0,7 | 1,16 | MH | NOMH | None | 90 | 0.7 | 0.55 |
| PT2 | 12 | F | M1E0S0T0 | CS | 4 | 109 | 0,6 | 0,814 | MH | NOMH | None | 119 | 0.55 | 0.44 |
| PT3 | 14 | M | M1E1S0T0C0 | CS | 3 | 67 | 1 | 0,394 | MH | NOMH | None | 77 | 0.87 | 0.39 |
| PT4 | 9 | M | M1E1S0T0C2 | CS | 7 | 98 | 0,59 | 0,218 | MH | NOMH | None | 97 | 0.6 | 0.183 |
| PT5 | 14 | M | M1E1S0T0C1 | CS, MMF | 6 | 82 | 0,9 | 1,4 | MH | NOMH | None | 98 | 0.76 | 0.726 |
| PT6 | 7 | F | M1E0S0T0C2 | CS, MMF | 6 | 130 | 0,45 | 0,525 | MH | NOMH | None | 119 | 0.50 | 0.136 |
| PT7 | 10 | M | M1E0S0T0C0 | CS | 5 | 114 | 0,6 | 0,083 | MH | NOMH | None | 124 | 0.55 | 0.059 |
| PT8 | 8 | M | M1E0S0T0C0 | CS | 4 | 103 | 0,64 | 0,4 | MH | NOMH | None | 94 | 0.71 | 0.164 |

Legend: PT: patient; M: male; F: female; CS: corticosteroids; MMF: micofenolate mofetil.MH: macroscopic hematuria; NOMH no macroscopic hematuria.
